# Homeopathy: does a teaspoon of honey help the medicine go down?

**DOI:** 10.3747/co.2007.150

**Published:** 2007-08

**Authors:** S.M. Sagar

Homeopathic remedies are undergoing a resurgence of popularity because the patient can administer them without a prescription, and they are alleged to have no adverse side effects. They are readily available in most drug stores and pharmacies, despite the fact that the theory of homeopathy is contrary to the theoretical foundation of every pharmacist’s training.

In some countries, patients are demanding that homeopathy be paid for through their national health insurance. Europe and Asia have a homeopathy tradition. In India, homeopathy is used as a treatment for cancer. In many other countries, it is used as part of a supportive care program for cancer patients, despite the lack of any credibility for its effect at a molecular level and just a few clinical trials that endorse its efficacy. Today, courses in homeopathy are even being offered at some institutions of higher learning.

Homeopathic remedies are prepared through a process called *potentization.* This involves a series of systematic dilutions and *succussions* (forceful shaking actions). Homeopathic practitioners allege that these actions eliminate chemical toxicity and enable the therapeutic effect. Homeopathic potencies are designated by a combination of a number and a letter (for example, 6X or 30C). The number refers to the number of dilutions that the tincture has undergone in series to prepare the remedy. The letter refers to the proportions used in each dilution of the series (the Roman numeral X means 10, and the Roman numeral C means 100) and the number of succussions that the vial of solution undergoes at each successive stage.

The law of infinitesimals in homeopathy states that dilution *increases* the curative power of homeopathic medications. This means that a 1-part-per-million solution of a substance is more medicinally powerful than a 1-part-per-thousand solution, which in turn has more curative power than a 1-part-per-hundred solution. In contrast, many modern drugs are ineffective at low doses, with their efficacy increasing with increasing dosage.

Many homeopathic remedies come in 30X or 200C dilutions. A substance diluted to 30X contains 1 part of the substance in 10^29^ parts water. A very important concept in chemistry is the Avogadro number, which is determined by X-ray diffraction of crystals. It has a value of 6.0221367 × 10^23^ as calculated by the International Council of Scientific Unions. Taking this constant into account, the limit imposed upon dilution—that is, the dilution that can be made without losing the original substance altogether—is 12C or 24X. A dilution of 30X is far outside this limit; to obtain even a single molecule of a substance in a solution diluted 10^30^ times, it would be necessary to drink almost *30,000 L* of the solution!

Samuel Hahnemann, the inventor of homeopathy, had his own explanation for the skeptic: his potentization–dynamization theory. The vigorous shaking causes the substance to leave behind a “spirit-like” essence that, although “no longer perceptible to the senses,” is nevertheless “remembered” by the water, and thus retains healing properties. Even keeping an open mind and accepting that, yes, these as yet-undetected forces may be at work in the water or sugar solution, it would seem likely that the water would retain a “memory” of everything inside the substance—not just the medicinal components, but also all the “contaminants” it has come into contact with. Each and every one of these bits—impurities and contaminants as well as the medicine itself—would make an imprint in the water. Hahnemann never mentioned anything about water being selective about what it remembers.

Similarly, it is logical to assume that the components in a substance are ignorant about their relative importance. What possible effects might the unwanted substances exert, and would those effects outweigh the medicinal effects?

Some homeopathic cures are marketed in pill form: lactose pills on which a single drop of infinitely dilute solution has been placed. How does this dilute drop convey the memory stored in the water to the lactose molecules? Even if subatomic fields govern the postulated “memories,” how can lactose in solid form “remember” information in the same way as water? And how does it convey that information to the cells in the body? Does it convey the information back to water first? Any possible evidence to support this idea has been repudiated.

Some natural health products are sold as homeopathic agents, despite the fact that they *do* contain detectable levels of pharmacologically active agents. A 2X–3X solution in homeopathic terms is paradoxically termed “low potency.” However, it may well be the limited dilution that is pharmacologically active. When analyzing a study, it is important to evaluate the exact content and degree of dilution. It is not inconceivable that a 2X dilution could have some pharmacologic activity. For example, Traumeel S (TRS, New York, NY, U.S.A.), an anti-inflammatory, is a complex mixture of natural health products termed homeopathic, but mainly diluted only to the 2X–3X level. A randomized controlled trial demonstrated that Traumeel S improves chemotherapy-induced mucositis 1. Currently it is being evaluated in a large randomized controlled clinical trial organized by the Children’s Oncology Group.

Are homeopathic remedies actually bad for you?

An ill person who goes straight to homeopathic cures before seeing a physician may delay life-saving treatment. In the case of cancer, early diagnosis is usually associated with improved prognosis. Delayed diagnosis and avoidance of evidence-based treatment may considerably worsen the prognosis. For non-serious health-related conditions, the patient *does* have the freedom of choice for homeopathy (although that choice would not be recommended).

Homeopathic remedies are nothing more than sugar and water. No evidence exists to suggest that water contains impregnated memory or that subatomic fields have ever affected anyone. Needless to say, as compared with most medication, homeopathic cures have no side effects—except possibly in the highly improbable case when diluted homeopathic medicine actually contains a molecule or two of the original substance.

The choice is entirely with the individual. Those who believe in the power of undetected subatomic fields may continue taking homeopathic medicine with an excellent placebo effect, but at a financial cost that cannot be ignored. Those who maintain faith in today’s science may continue to see their physician and receive conventional medication proven in clinical trials, rather than in succussion. However, if you choose to save money and avoid side effects, a teaspoon of honey (composed mainly of sugar and water) may be more attractive—unless, of course, you are allergic to bees or pollen.

In the article that follows, Professor Edzard Ernst, md phd frcp frcped, who holds the Laing Chair in Complementary Medicine at the Peninsula Medical School, University of Exeter, U.K., reviews the published evidence for the role of homeopathy in cancer care.
